# Development of a novel sandwich-structured composite from biopolymers and cellulose microfibers for building envelope applications

**DOI:** 10.1038/s41598-023-49273-0

**Published:** 2023-12-11

**Authors:** Masoud Dadras Chomachayi, Pierre Blanchet, Atif Hussain, Simon Pepin

**Affiliations:** 1 Department of Wood and Forest Sciences, Laval University, Quebec City, QC G1V 0A6 Canada; 2 Department of Materials Engineering, University of British Columbia, Vancouver, BC V6T 1Z4 Canada

**Keywords:** Engineering, Materials science, Biomaterials, Polymer chemistry

## Abstract

A novel sandwich-structured composite was developed from the surface layers of polyhydroxyalkanoate (PHA) and the interlayer of polylactic acid (PLA)/cellulose microfibers (CMF) composite. Moreover, CMF was chemically modified via a sol–gel process to improve the compatibility between the natural reinforcement and the polymer matrix. According to the obtained results, the modified CMF exhibited a highly hydrophobic characteristic (contact angel value of approximately 118°), and they were homogeneously dispersed in the PLA matrix. The results of the thermogravimetric analysis indicated that the sandwich composites reinforced with the modified CMF showed improvement in thermal stability. Regarding the mechanical properties, the incorporation of the natural reinforcement into sandwich composites increased the values of tensile modulus and strength of materials. The water vapor permeability of sandwich composites increased with the addition of untreated fibers; however, the composites reinforced with the modified CMF showed superior barrier performance than that of untreated CMF. In addition, a durability test was performed to determine the effect of accelerated aging on the properties of sandwich composites. The results demonstrated that the mechanical and barrier properties of composites incorporated with untreated CMF decreased after the accelerated aging, whereas the composites reinforced with the modified CMF experienced the least change.

## Introduction

The building envelope is an essential part of buildings that is responsible to protect the building occupants from exterior environmental impacts such as sun, rain, snow, wind, and pollution. The building envelope is a multilayer passive element that has an important effect on the energy efficiency of buildings while maintaining the health and comfort of the building's residents^1^. Air leakage, heat transfer, and moisture diffusion are some of the significant functions of building envelopes^2^. In cold climate countries, the mass concentration of water vapor in the interior side of the building is usually higher than in the exterior environment, thereby leading to the migration of moisture through the building walls^3^. As a consequence, the diffused moisture can encourage mold growth, reduce the effectiveness of insulation, and deterioration of building envelope materials^4^. In this regard, a vapor barrier/retarder membrane should be installed on the interior side of the building (warm side) to control the transmission of water vapor through the building walls^5^.

There are several types of barrier membranes currently used in the building industry such as plastic sheeting and rigid foam insulation. Among these materials, polymers are increasingly used in the building envelope application due to their unique. Polyethylene (PE) extruded sheets are one of the most prominently vapor barrier/retarder membranes used in the market. Nevertheless, PE is a synthetic petrol-derived polymer that has a relatively high embodied energy and carbon footprint, and its excessive utilization is harmful to the environment^6^. Nowadays, the regulation on the usage of fossil-based plastics has been tightened globally as a front control strategy^7^. Such global trends led to an increase in demand for materials derived from renewable resources that have the potential to be considered as an alternative to conventional fossil-based plastics.

Polyhydroxyalkanoate (PHA) and polylactic acid (PLA) are the most important biopolymers in the plastic industry^8^. PHA is a natural thermoplastic biopolymer that is used in several applications such as food packaging and biomedical applications given its excellent gas/vapor barrier property and biocompatibility. However, it is still a challenge to manufacture a product made of 100% PHA because of the high manufacturing cost and its processing instability. To make PHA more industrially applicable and feasible for commercial applications it can be blended with other renewable materials and reinforcements. Recently, PLA has received great attention and become one of the most economical biopolymers in industrial applications. It has several desirable properties including excellent processability, physicochemical properties, and reasonable price^9^. Despite its interesting properties, it has some drawbacks that limit its applications for engineering purposes. These disadvantages are included its poor thermal stability, poor toughness, and slow crystallization rate^10^. These issues could be addressed upon the incorporation of a natural reinforcement (e.g., wood flour or cellulose fibers) into composites^11^. In these materials, named wood-plastic composite, the natural filler not only has a reinforcement effect but also reduces the carbon footprint and the weight of products^12^.

Recently, several studies reported the utilization of renewable cellulose-based fillers in polymer composites^13^. Cellulose microfibers (CMF) are an ideal candidate to be used as a reinforcement in polymer composites. CMF has several interesting properties including abundance, non-toxicity, and good mechanical properties. Moreover, CMF offers a high surface area for interaction with the polymer matrix, providing superior characteristics compared to large-scale cellulosic fibers in polymer composite applications. The main challenge in the production of fibers reinforced composites is achieving an appropriate dispersion of fibers in the polymer matrix^6^. CMF has hydrophilic nature due to the presence of many hydroxyl (OH) groups in its structure making it incompatible with a non-polar polymer. In addition, CMF absorbs moisture from the surrounding, resulting in the swelling of fibers and the formation of internal stresses between the polymer matrix and fibers. Therefore, the chemical surface modification of CMF should be conducted to convert its hydrophilicity to hydrophobic characteristic^14^. There are various methods for the modification of CMF, e.g., esterification^15^, oxidation/amidation^16^, alkalization^17^, surfactant^18^, plasticizer^19^, polymer grafting^20^, silanization^21^. The sol–gel technique is a highly versatile method for modifying natural reinforcements^22,23^. This method is based on hydrolysis and in-situ condensation of an inorganic precursor on the surface of cellulosic fibers^24,25^. Tetraethylorthosilicate^26^, methyltrimethoxysilane^7^, and hexadecyltrimethoxysilane^27^ are some of the silane-based precursors previously used for the modification of natural reinforcements^28,29^.

The present study aims to develop a novel sandwich-structured barrier membrane from bio-based materials. PHA was selected for the surface layers of membranes owing to its excellent vapor barrier properties. Despite PLA's susceptibility to cold climates, it was selected as the interlayer in the sandwich composites, incorporating up to 30 wt% cellulose microfibers for environmental and economic benefits. To improve the dispersibility of the hydrophilic fibers in the PLA matrix, CMF was modified with TEOS and HDTMS via the sol–gel process. Moreover, the prepared sandwich composites were characterized in terms of their morphology, thermal stability, mechanical properties, and vapor barrier performance. Additionally, a durability test was performed to investigate the effect of accelerated aging on the mechanical and barrier properties of materials.

## Materials and methods

### Materials

Polylactic acid (PLA) (4043D) with a melt flow index (MFI) of 6 g/10 min (at 210 °C, 2.16 kg) was obtained from NatureWorks (Minnetonka, MN, USA). The extruded polyhydroxyalkanoate (PHA) pellets (REGEN™) with a MFI of PHA is 3–5 g/10 min (at 190 °C, 2.16 kg) were purchased from Bosk Bioproducts (Quebec, QC, Canada). The specific gravity and melting temperature of PHA are 1.24 g/cm^3^ and 170 °C, respectively. Cellulose microfibers (CMF), commercially named cellulose filaments, were provided by Kruger Biomaterials Inc. (Montreal, QC, Canada). The CMF was derived from northern bleached softwood kraft (NBSK) pulp. The CMF contains a certain proportion of cellulose nanofibrils, and the average diameter and bulk density of CMF are 8 ± 1 μm and 633 kg/m^3^, respectively. The commercial polyethylene (PE) barrier membrane (6 mil thickness) was purchased from Balcan Plastics Inc. (Montreal, QC, Canada). Moreover, Chloroform (CHCl_3_), ethanol, ammonium hydroxide (NH_4_OH), tetraethylorthosilicate (C_8_H_20_O_4_Si) (TEOS), and hexadecyltrimethoxysilane (C_19_H_42_O_3_Si) (HDTMS) were supplied from Sigma-Aldrich (Oakville, ON, Canada).

### Modification of CMF with sol–gel method

**Figure 1:**
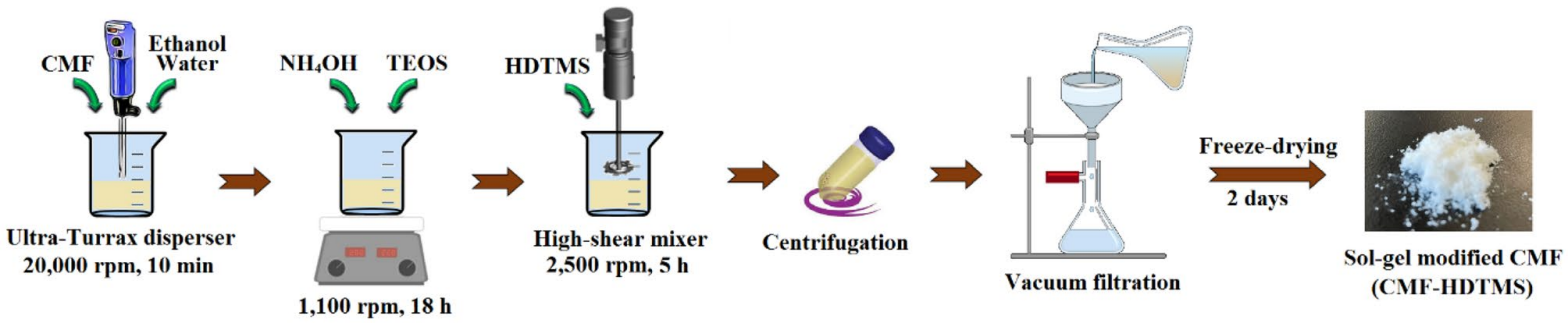
Schematic illustration of the modification procedure of CMF via the sol–gel process.

The surface modification of CMF using TEOS and HDTMS was conducted in a continuous sol–gel process. Firstly, 5 g of dried CMF was added to a mixture of 45 ml deionized water and 425 ml ethanol, and the suspension was mixed in an Ultra Turrax disperser at 20,000 rpm for 10 min. Then, 7.5 ml of NH_4_OH (base catalyst) was added to the mixture while stirring at 1100 rpm for 2 h. For the deposition of SiO_2_ nanoparticles onto CMF’s surface, 45 ml of TEOS (hydrophilic precursor) was added dropwise to the CMF suspension, and constant and moderate mechanical stirring was kept at 1100 rpm for 18 h (reaction time). Afterward, 45 ml of HDTMS (hydrophobic precursor) was added dropwise to the mixture while mixing in a high-shear mixer at 2500 rpm for 5 h. At the end of the reaction, the mixture was centrifuged at 1000 rpm for 5 min and then vacuum filtered, followed by washing three times with ethanol. Finally, the obtained hydrophobically CMF was freeze-dried for 2 days and then ground in a grinder (Retsch ZM100, Germany) to obtain the modified CMF powder. Schematic image of the modification procedure of CMF via the sol–gel process is presented in Fig. 1:

### Preparation of sandwich-structured membranes

#### PLA/CMF interlayer composites

The PLA/CMF composites were prepared through the solvent casting method. Priorly, PLA pellets and CMF were vacuum-dried overnight at 70 °C to remove moisture. The desired amount of PLA was dissolved into 100 ml CHCl_3_ under stirring at 600 rpm for 2 h at room temperature. Then, the PLA solution was mixed with different content of untreated CMF or modified fibers (CMF- HDTMS) (5, 10, 20, and 30 wt%). Subsequently, the solution was sonicated for 10 min at room temperature, followed by stirring at 1000 rpm for 1 h. Finally, the prepared solutions were cast onto a glass petri dish and dried by solvent evaporation at room temperature for 2 days in a fume hood. It should be noted that in each formulation, the total weight of all composites’ components was the constant value of 5 g.

#### PHA sheets and layer-by-layer composites

Priorly, PHA pellets were vacuum-dried overnight at 70 °C to remove moisture. For the preparation of PHA sheets, PHA pellets were compression molded into sheets with a thickness of ~ 100 μm (13 × 13 cm) in a steam injection press (two plates metal molds) (Dieffenbacher, Alpharetta, GA, USA), beginning with a melting step at 180 °C for 5 min with no pressure, followed by pressing at 5 MPa for 2 min. Finally, the samples were cooled in a cold press (two plates metal molds), SPX hydraulic pumps (Power Team, Rockford, IL, USA) using a pressure of 10 MPa for 5 min at room temperature.

**Figure 2:**
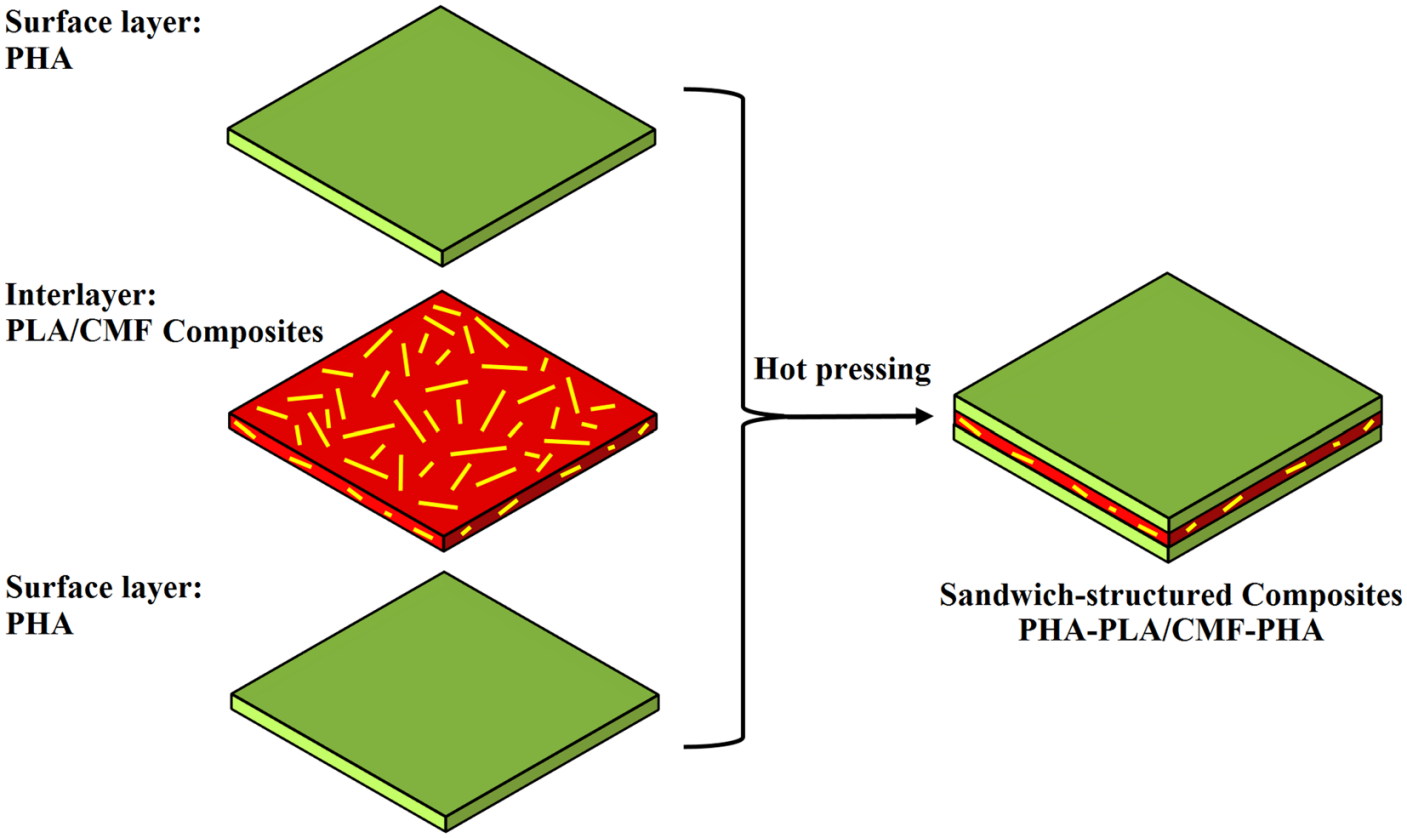
Schematic image of the preparation of sandwich-structured composites of PHA-PLA/CMF-PHA.

The sandwich-structured composites were fabricated by the compression molding technique. In this regard, the prepared PHA sheets (top/bottom layers) and PLA/CMF composites (interlayer) were compression molded into sheets with a thickness of 300 μm (13 cm × 13 cm) in the steam injection press at 180 °C, beginning with a melting step for 2 min with no pressure, followed by pressing at 5 MPa for 2 min. Then, the sheets were cooled in the cold press using a pressure of 10 MPa for 5 min. The sandwich-structured composites with the interlayer of PLA/CMF composites incorporated with untreated CMF or modified fibers (CMF-HDTMS) were coded as PHLACx and PHLACSx, respectively (where x indicates the content of natural reinforcement). The formulation of all samples is given in Table 1. Moreover, the schematic image of the preparation of PHA-PLA/CMF-PHA sandwich composites is given in Fig. 2. Before the characterization, all prepared samples were vacuum-dried at 40 °C for 4 days to remove the remaining moisture and then stored in a zipped airtight bag.

**Table 1:** The formulation of sandwich-structured composites of PHA-PLA/CMF-PHA.

Top/bottom layers	Interlayer	Denotation
PHA	PLA	PHLA
PHA	PLA95/C5	PHLAC5
PHA	PLA90/C10	PHLAC10
PHA	PLA80/C20	PHLAC20
PHA	PLA70/C30	PHLAC30
PHA	PLA95/CS5	PHLACS5
PHA	PLA90/CS10	PHLACS10
PHA	PLA80/CS20	PHLACS20
PHA	PLA70/CS30	PHLACS30

### Characterization techniques

#### Scanning electron microscope (SEM)

The scanning electron microscope (SEM), JEOL 6360LV (Hitachi, Japan), was used to investigate the morphology of CMF (before and after the modification), PLA/CMF composites (interlayer), and sandwich composites. To study the cross-section morphology of sandwich composites, the samples were first immersed in liquid nitrogen for 5 min and then fractured immediately. Before observation, the surface of the samples was coated with a thin layer of gold using an EMS 950× sputter coater (Hatfield, PA, USA). The SEM test was performed under vacuum conditions and an accelerating voltage of 15 kV.

#### Contact angle measurement

The effect of sol–gel modification on the hydrophobic properties of CMF was studied by the contact angle measurement. In this regard, 0.1 g of dried natural reinforcements (untreated CMF or CMF-HDTMS) was compressed at 30,000 kN for 5 min into a disc of 13 mm in diameter using the MTS Alliance RT/5 (Eden Prairie, Minnesota, USA). Three discs with a thickness of 0.5 mm were prepared for each natural reinforcement. Thereafter, the contact angle of a DI water droplet was measured with the contact angle goniometer, FTA200 (Portsmouth, VA, USA). The contact angle values were determined from the instrument’s software (FTA32) and the average values of contact angles versus time were reported over 10 s.

#### Fourier‑transform infrared spectroscopy (FT-IR)

A Fourier transform infrared spectroscopy (FT-IR), Bruker INVENIO® R (Billerica, MA, USA) was carried out to study the chemical structure of CMF (before and after the modification) and PLA/CMF composites (interlayer). The FT-IR scans were collected in absorbance mode in the wave number range of 4000–400/cm with a spectral resolution of 4/cm.

#### Thermogravimetric analysis (TGA)

The thermal stability of materials including CMF (before and after the modification), neat polymers (PE, PHA, PLA), and sandwich composites was investigated using the thermogravimetric analysis (TGA), TGA/DSC 3+ Mettler Toledo instrument (Mississauga, ON, Canada). About 10 mg of natural reinforcement and polymer composites were heated from 30 to 950 °C and 30 to 600 °C, respectively, at a heating rate of 10 °C/min and under nitrogen-purged atmospheres (at 50 ml/min).

#### Differential scanning calorimetry (DSC)

The differential scanning calorimetry (DSC) analysis was utilized to characterize the thermal properties of the natural reinforcement before and after the sol–gel modification. The DSC test was performed using a DSC Mettler Toledo 822/e (Columbus, OH, USA). Each sample (~ 10 mg) was heated at a rate of 10 °C/min, from 25 to 400 °C, under nitrogen-purged at 50 ml/min.

#### Tensile test

The mechanical properties of materials including the neat polymers (PE, PHA, and PLA) and sandwich-structured composites were investigated by means of uniaxial tensile test based on ASTM D882 standard^30^. Each specimen with a thickness of 300 μm was cut into a rectangular shape (10 cm × 1 cm) using a laser machine. The tensile test was performed at 500 N and a cross-head rate of 12.5 mm/min using the MTS QTest/5 (Eden Prairie, Minnesota, USA). The analysis was repeated in triplicates (n = 3) for each sample and the average values of tensile strength (MPa), Young’s modulus (MPa), and elongation at break (%) were reported.

#### Water vapor transmission rate (WVTR)

The water vapor permeability of neat polymers (PE, PHA, PLA) and sandwich composites were studied using the water vapor transmission rate (WVTR) test based on the ASTM E96 standard (methods A and B)^31^. In this regard, each specimen with a thickness of 300 μm was placed in a cup with an internal diameter of 6 cm. To avoid the migration of water vapor through boundaries, the surrounding area of the specimen was sealed with silicone sealant. In method A (desiccant method), the cup was filled with approximately 102 g of dried CaCl_2_ desiccant to a level 6 mm from the specimen. In method B (water method), the cup was filled with distilled water to a level of 19 ± 6 mm from the specimen. The relative humidity inside of the permeability cup in methods A and B are 0% and 100%, respectively. Each sample cup was initially weighed (W_0_) and then placed in a controlled temperature/humidity chamber (23 °C/50% RH) for three weeks. The weight of each cup was measured every day (W_n_) to calculate the amount of gained water or evaporated/escaped water in methods A and B, respectively. Then, the graphs of mass change (W_n_-W_0_) versus time were plotted and the value of WVTR was obtained using the following equation:

1 $$ {\text{WVTR}} = \frac{{\left( {{\text{G}}/{\text{t}}} \right){ }}}{{\text{A }}}\;\;\left( {{\text{g}}/{\text{h}}\;{\text{m}}^{{2}} } \right) $$

where *G* is the steady-state weight change (g), *t* is time (h), and *A* is the area of the permeability cup (0.00255 m^2^). Moreover, the value normalized WVTR was calculated by eliminating the thickness factor of samples as follows:

2 $$ {\text{Normalized }}\;{\text{WVTR}} = {\text{WVTR}} \times d $$

where *d* is the thickness of each sample (m). The WVTR test was performed in triplicates (n = 3) for each sample and the average values of normalized WVTR were reported.

#### Accelerated aging

In this study, accelerated aging was studied according to the ASTM D1183 standard^32^ to simulate magnified interior building conditions (low and high humidity) by exposing the samples to cyclic variations of moisture/temperature (Table 2). The aging process was performed in an environmental test chamber (Envirotronics, Grand Rapids, MI, USA). As can be seen in Table 2, more time was allotted to humid conditions. After the 10 cycles of accelerated aging, the mechanical and barrier properties of the samples were examined.

**Table 2:** The test conditions of one cycle of accelerated aging.

Test conditions	Period (h)	Temperature (°C)	Relative humidity (%)
High humidity	72	23 ± 1	90
Low humidity	24	48.5 ± 1	25

## Results and discussion

### Characterization of CMF

**Figure 3:**
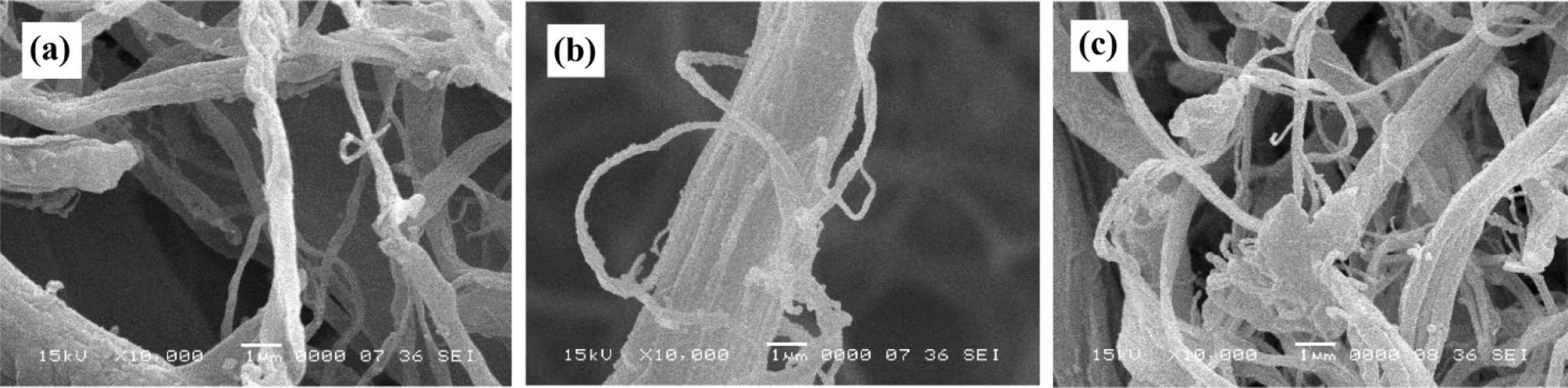
SEM images of: (**a**) untreated CMF, (**b**) CMF-TEOS, and (**c**) CMF- HDTMS.

The surface morphology of CMF before and after the modification was examined via the SEM analysis, and the results are presented in Fig. 3. As can be observed in Fig. 3a that the untreated CMF had a smooth surface, and the average diameter of fibers was 8 ± 1 μm. Figure 3b exhibits the morphology of CMF after the modification via TEOS (hydrophilic precursor). This figure indicates that the spherical silica nanoparticles (SiO_2_) with a diameter of 130–150 nm were formed onto the fibers, making the CMF’s surface rougher. Indeed, the nanoparticles were derived from the hydrolysis and nucleation of TEOS precursor onto the CMF^27^. Previous studies demonstrated that the surface roughness of cellulose fibers increased after the treatment with silica hydrosol^33^. For instance, Raabe et al., treated cellulose fibers with TEOS using a sol–gel process, and the results exhibited that SiO_2_ nanoparticles with a diameter of ~ 150 nm were formed onto the fibers. They also investigated the effect of the modification reaction time and TEOS content on the properties of cellulose fibers. According to the results, the best process condition was a reaction time of 18 h and TEOS content of 8.4 g/g (per g of cellulose fibers) which provided a uniform SiO_2_ nanoparticles coating on the surface of fibers^26^.

Figure 3c shows the SEM images of SiO_2_-coated CMF after the sol–gel process via HDTMS. It is interesting to mention that by the deposition of long-chain alkylsilanes of HDTMS onto the SiO_2_-coated CMF, the spherical shape of nanoparticles was less defined (Fig. 3c). It could be interpreted that HDTMS filled the interspaces between SiO_2_ nanoparticles^33^. It can be expected that the presence of hydrophobic long-chain alkylsilanes onto the CMF’s surface could prevent the entanglement among fibers^29^. Similarly, Jo et al.^7^ reported that the double salinization of cellulose nanocrystals (CNCs) via TEOS and methyltrimethoxysilane (MTMS) changed the surface characteristics of CNCs from hydrophilic to hydrophobic, thereby avoiding the agglomeration of nanoparticles to larger particles.

**Figure 4:**
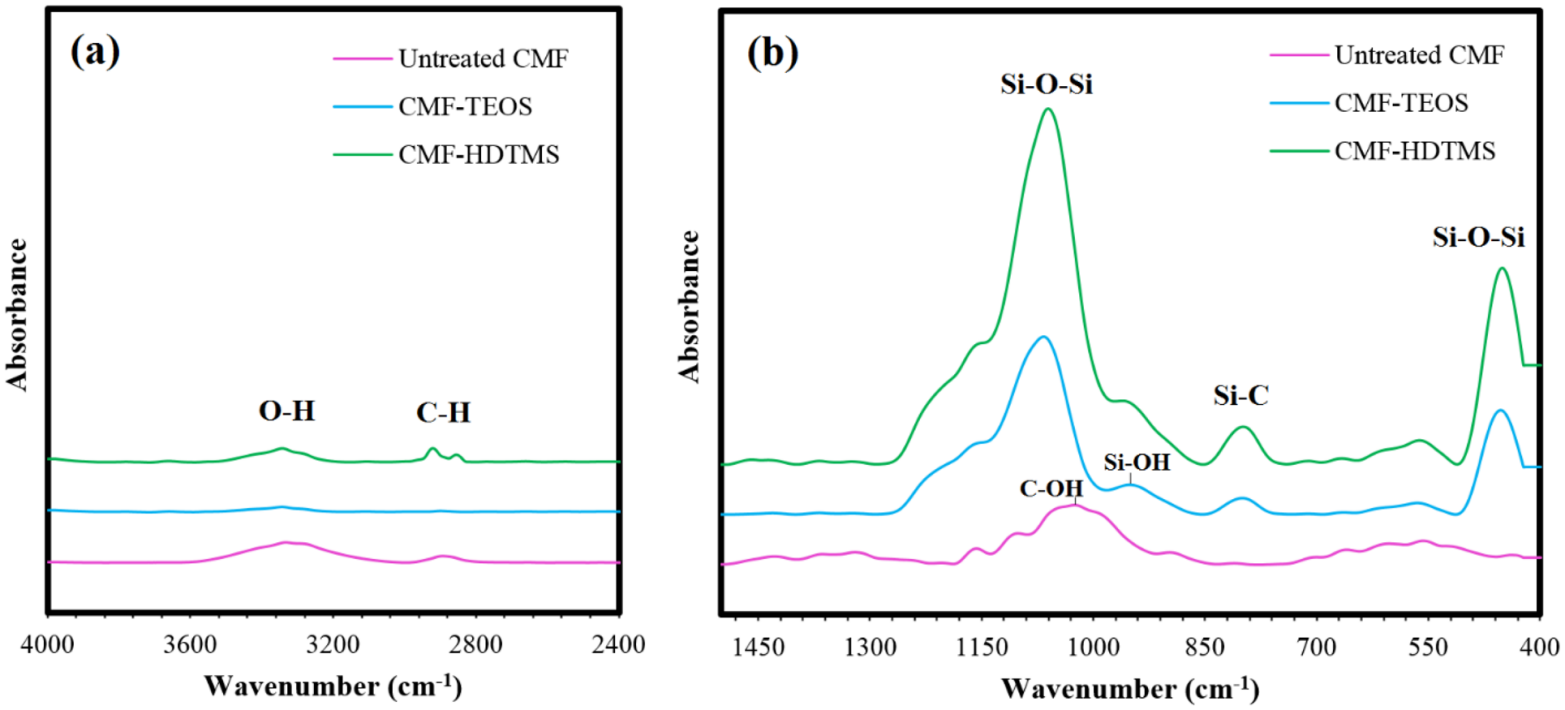
FT-IR spectra of untreated CMF, CMF-TEOS, and CMF-HDTMS: (**a**) 4000–2400/cm and (**b**) 1500–400/cm wavenumbers.

The FT-IR analysis was used to determine the chemical structures of CMF before and after the modification via the sol–gel process, as indicated in Fig. 4. In the spectra of untreated CMF, the wide band from 3100 to 3500/cm is assigned to the OH stretching vibration of cellulose molecules. Moreover, the peaks located at 2888, 1090, and 1028/cm are corresponded to the CH, and C–OH stretching of secondary and primary alcohols of cellulose, respectively^6^. Regarding the spectra of CMF-TEOS, the intensity of OH and CH methyl groups of cellulose significantly reduced, while three peaks appeared at 450/cm (Si–O–Si stretching), 800/cm (Si–C vibration), and 1084/cm (Si–O–Si stretching), which are attributed to the main typical bands of silica. Moreover, the peak at 950/cm is related to the OH end groups of SiO_2_ nanoparticles deposited on the surface of fibers. The presence of silica peaks suggests the successful silanization of CMF using TEOS^7^. After the deposition of HDTMS onto the SiO_2_-coated CMF, the silica peaks at 450, 800, and 1084/cm were pronounced, whereas the intensity of the Si–OH band at 950/cm decreased. In addition, two absorption peaks appeared at 2850 and 2920/cm, which corresponded to the C–H stretching vibrations of –CH_3_ and –CH_2_ of long-chain alkylsilanes of HDTMS, respectively^27^. Therefore, it can be concluded that the deposition of HDTMS onto the CMF’s surface was induced by the reaction between the alkylsilanes of HDTMS and the OH groups of SiO_2_ nanoparticles, implying the hydrophobic modification of CMF.

**Figure 5:**
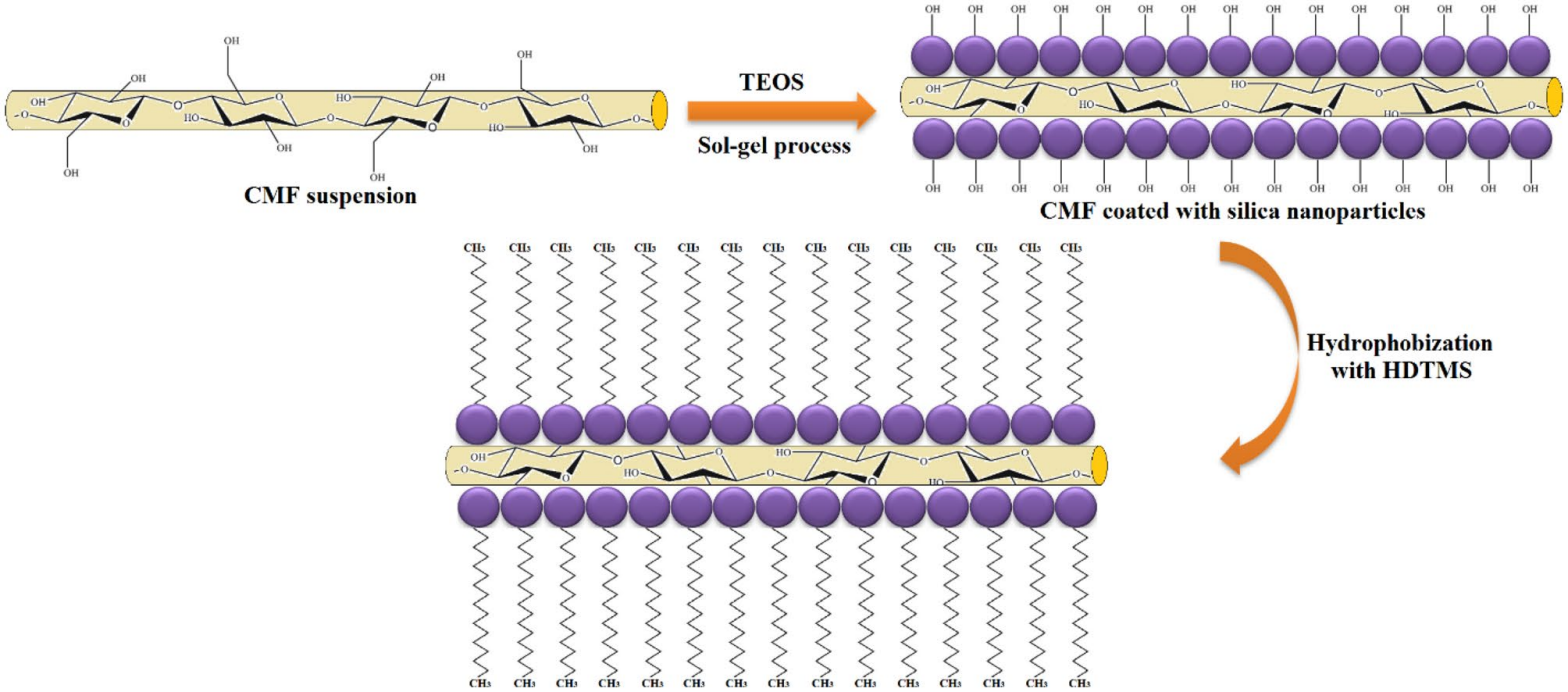
Schematic of modification of CMF via the sol–gel process.

The schematic illustration of the sol–gel modification of CMF with TEOS and HDTMS is presented in Fig. 5. Briefly, in the first stage, the TEOS molecules were hydrolyzed in an alkaline condition followed by their in-situ condensation on the surface of fibers. Afterward, the nucleation of silica precursor formed the spherical growth of SiO_2_ nanoparticles onto the CMF’s surface. Indeed, the OH groups of cellulose play an important role to adhere SiO_2_ nanoparticles by forming interfacial C-O-Si bonds with the Si–OH groups of TEOS. It should be noted that the surface of SiO_2_-coated CMF is still hydrophilic due to the presence of OH groups of SiO_2_ nanoparticles. In the second step, the SiO_2_-coated CMF was treated via HDTMS to impart hydrophobic characteristics to CMF. The Si-OCH_3_ groups of HDTMS are very reactive and can be converted to the Si–OH groups; therefore, the HDTMS molecules were hydrolyzed to alkylsilane in the alkaline medium. Meanwhile, the dehydration reaction carried out between the alkylsilane and the surface OH groups of SiO_2_ nanoparticles, led to the formation of Si–O–Si bonds. As a result, the hydrophobic long-chain of alkylsilanes was deposited on the surface of fibers.

**Figure 6:**
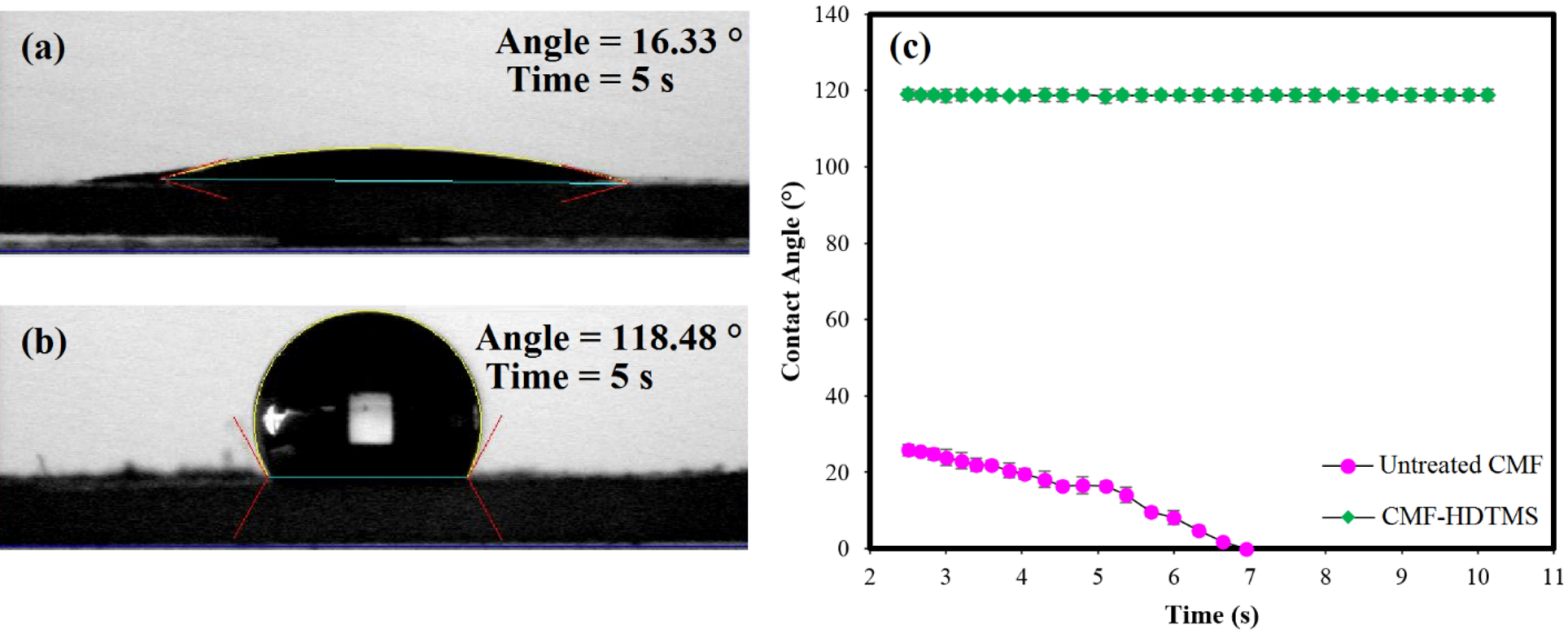
Contact angle results of: (**a**) untreated CMF and (**b**) CMF-HDTMS after 5 s. (**c**) Contact angle values of natural reinforcements versus time.

The effect of sol–gel modification on the hydrophobicity of CMF was evaluated by the contact angle measurement, and the results are provided in Fig. 6. The contact angle of untreated CMF after 5 s was 16.33° (Fig. 6a), whereas the value increased to 118.48° (Fig. 6b) after the modification. Figure 6c demonstrates that the contact angle values of CMF-HDTMS were remarkably higher than that of untreated CMF. Moreover, the contact angle value of untreated CMF decreased over time, and the water droplet completely spread on the surface of fibers after 7 s. The results reveal the hydrophilic nature of CMF, attributed to the abundant hydroxyl groups present on its surface^34^. On the other hand, the CMF-HDTMS showed a constant contact angle value of ~ 118° over time, suggesting a highly hydrophobic surface of fibers after the treatment. This is attributed to the formation of SiO_2_ nanoparticles on the CMF surface creates several cavities and interspaces between nanoparticles, which is one of the preconditions for making a hydrophobic surface^27^. In addition, the introduction of the hydrophobic long-chain of alkylsilanes of HDTMS onto the SiO_2_-coated CMF imparts hydrophobic character to the natural reinforcement. Similarly, a previous study also demonstrated that the double silanization of CNCs using TEOS and MTMS significantly enhanced the contact angle value from 23.2° to 150.4° (superhydrophobic surface)^7^.

**Figure 7:**
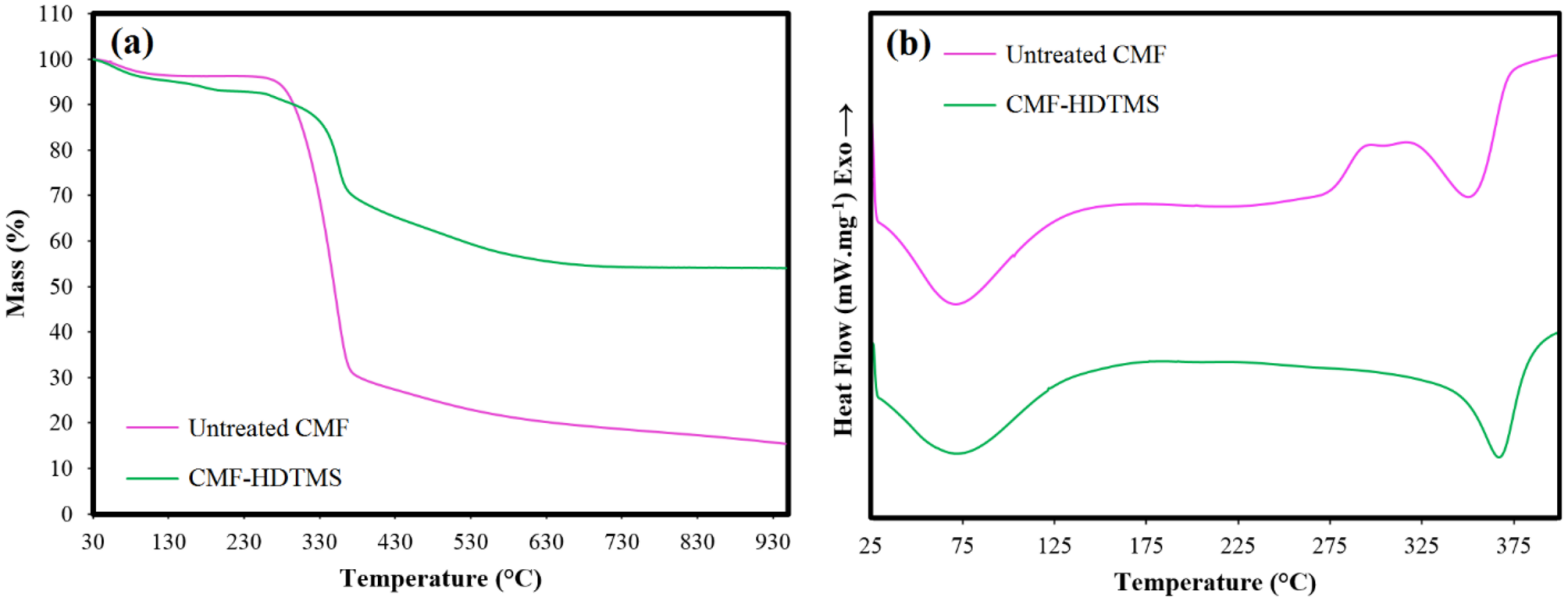
(**a**) TGA and (**b**) DSC first heating curves of untreated CMF and CMF-HDTMS.

Figure 7a presents the TGA curves of CMF before and after the modification via the sol–gel process. In the TGA thermograms, the thermal decomposition below 150 °C is attributed to the sample dehydration. As shown in Fig. 7a, both untreated CMF and CMF-HDTMS were thermally decomposed in a single step from 240 to 380 °C. Moreover, the results demonstrate that the CMF-HDTMS has a higher value of thermal stability than the untreated CMF in the temperature range over 330 °C. For instance, the maximum degradation temperature (T_max_) of untreated CMF (349.8 °C) shifted towards a higher temperature (353.9 °C) after the modification. Similarly, Raabe et al.^26^ reported that the deposition of SiO_2_ nanoparticles on the surface of cellulose fibers improved the thermal stability of the natural reinforcement. The remaining weight percentage (residual mass) of CMF-HDTMS at 950 °C was 54.1%, which is far higher than that of untreated CMF (15.4%). This might be due to the Si inorganic species formed on the surface of fibers that protect them from the thermal degradation^26^. The DSC first heating curves of CMF before and after the modification are given in Fig. 7b. The thermal decomposition temperature (T_d_) of untreated CMF and CMF-HDTMS were 340.1 and 373.2 °C, respectively. The results are in line with TGA results, implying that the sol–gel modification improved the thermal stability of CMF.

### Characterization of sandwich-structured membranes

#### SEM test

**Figure 8:**
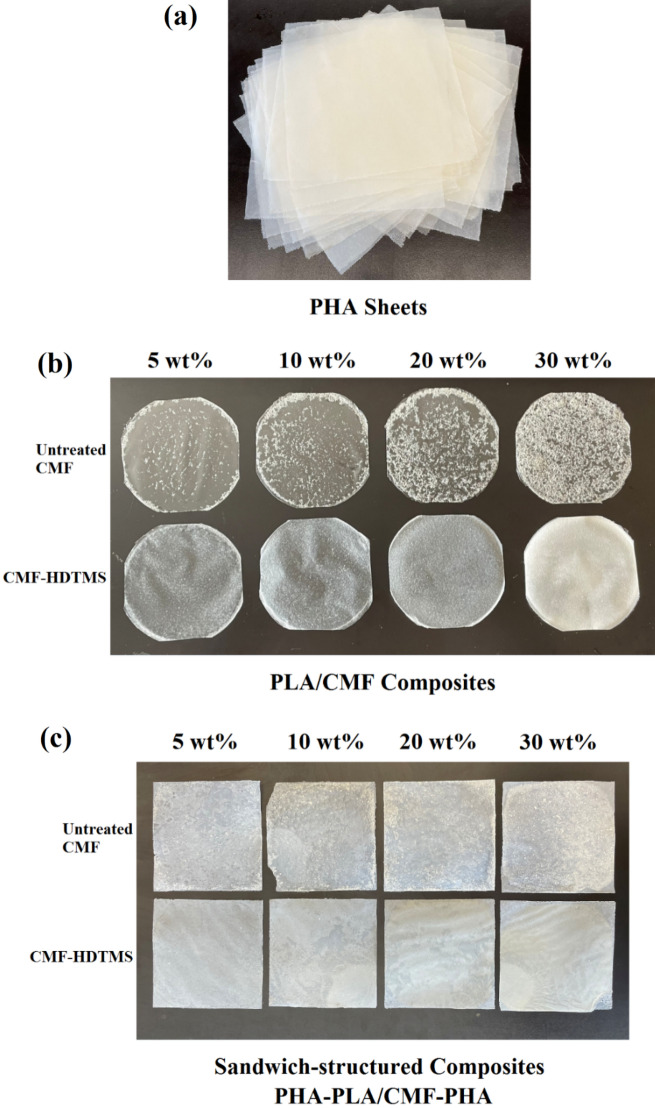
Images of (**a**) PHA sheets, (**b**) PLA/CMF composites, and (**c**) sandwich-structured composites.

**Figure 9:**
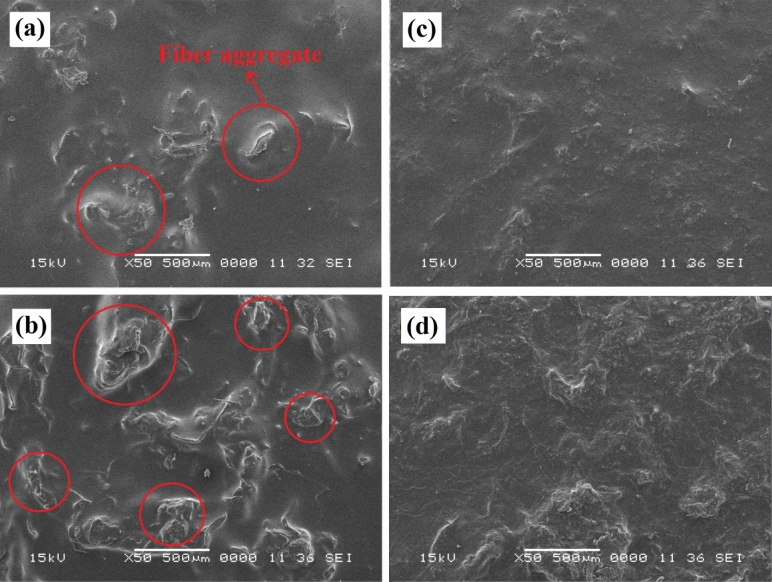
SEM images of the top surface of: (**a**) PLA95/C5, (**b**) PLA90/C10, (**c**) PLA95/CS5, and (**d**) PLA90/CS10.

The photographic images of prepared samples including the neat PHA sheets, interlayer composites (PLA/CMF), and sandwich-structured composites (PHA-PLA/CMF-PHA) are given in Fig. 8. It can be visually distinguished from Fig. 8b that the untreated CMF showed a poor dispersion in PLA, while the modified fibers (CMF-HDTMS) dispersed homogeneously in the polymer matrix. Additionally, the SEM technique was conducted to study the structural morphology of PLA-based composites, and the results are provided in Fig. 9. The SEM images of PLA/CMF composites incorporated with 5 and 10 wt% of untreated CMF are presented in Fig. 9a and b, respectively. As can be observed, the untreated CMF was agglomerated in the non-polar PLA matrix (shown by the red circle). Similarly, a previous study reported that cellulose fibers showed a poor dispersion in the PLA matrix, which is attributed to the strong interactions between the free OH groups of fibers and also a low thermodynamic affinity between the natural reinforcement and biopolymer^20^. On the other hand, the morphological images of PLA composites reinforced with 5 and 10 wt% of CMF-HDTMS (Fig. 9c and 9d) indicate an excellent dispersion of fibers in the PLA matrix, and there are no visible fiber aggregates in the structure. It can be deduced that the compatibility of CMF and PLA improved thanks to the modification of fibers via the sol–gel process.

**Figure 10:**
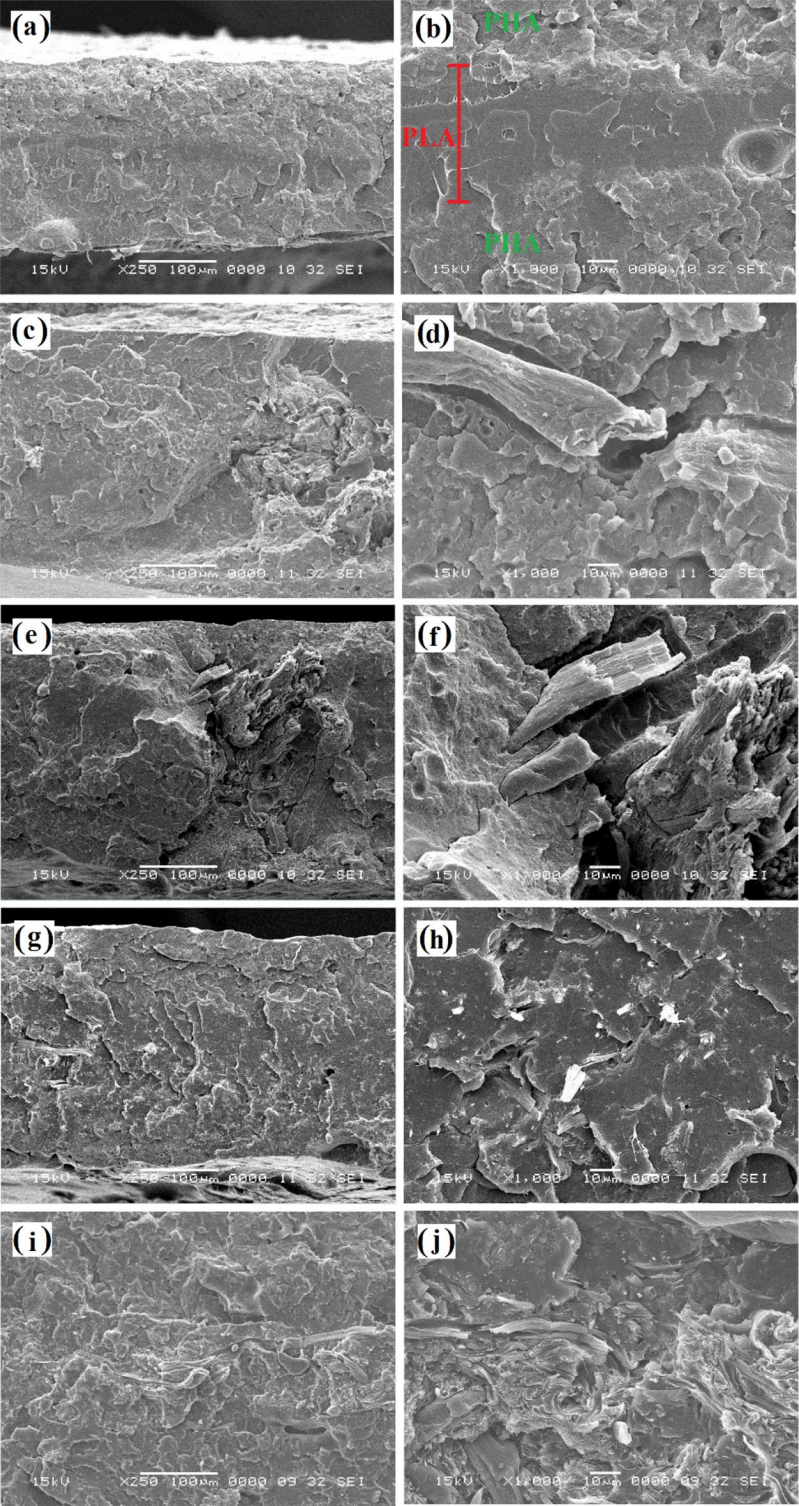
SEM images of the freeze-fractured surface of: (**a** and **b**) PHLA, (**c** and **d**) PHLAC5, (**e** and **f**) PHLAC10, (**g** and **h**) PHLACS5, and (**i** and **j**) PHLACS10.

The SEM images of the cross-section (freeze-fractured surface) of PHLA sandwich composites were provided in Fig. 10. As can be seen in Fig. 10a and b, the PLA interlayer with a smooth microstructure was surrounded by the surface layers of PHA having a rough and irregular topography. Moreover, there is a good attachment at the interface of PHA and PLA layers, indicating a proper interfacial adhesion between the two polymers. Regarding the cross-sectional SEM images of PHLAC5 (Fig. 10c and d) and PHLAC10 (Fig. 10e and f), the presence of voids around the fiber aggregates reveals a poor interfacial adhesion between the untreated CMF and PLA. Interestingly, the morphological images corresponding to the PHLACS5 (Fig. 10g and h) and PHLACS10 (Fig. 10i and j) illustrate that the presence of voids and agglomeration of fibers were nearly invisible in these composites. These observations suggest an enhancement in the dispersibility and interfacial adhesion of CMF-HDTMS within the polymer matrix. It can be expected that the hydrophobic modification of CMF with HDTMS (via the sol–gel process) and the introduction of long-chain alkylsilanes onto the surface of fibers can effectively improve the thermodynamic affinity between the natural reinforcement and non-polar polymer matrix. The results are in good agreement with a previous study reported by Jo et al.^7^ that the sol–gel modification of CNCs via the TEOS and MTMS improved the compatibility of CNCs and PHA polymer matrix. It is worth noting that the presence of voids and fiber aggregates in composites provides a preferential pathway for the migration of water vapor molecules through the materials, which dramatically has a negative impact on the barrier performance of composites^35^. The obtained results suggest that the sol–gel modification of CMF can be considered an effective method for fabricating homogenous fibers reinforced polymer composites.

#### FT-IR test

**Figure 11:**
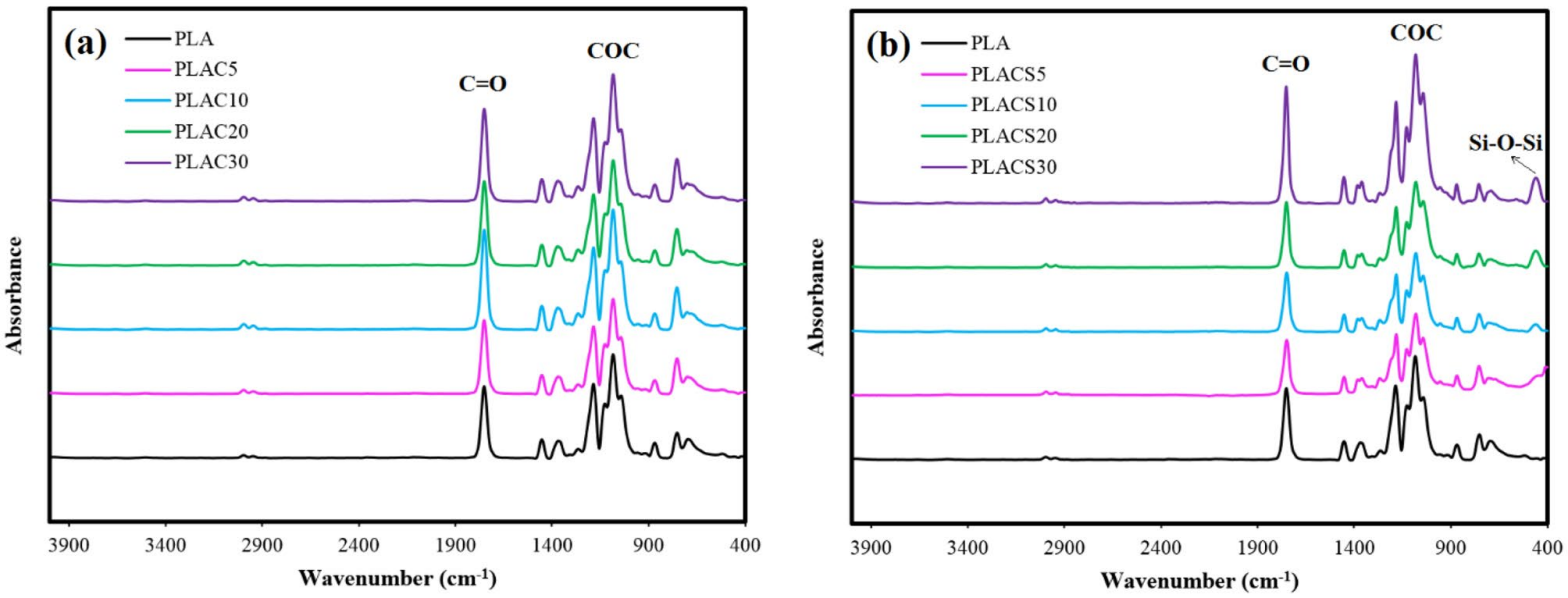
FT-IR spectra of PLA-based composites incorporated with (**a**) untreated CMF and (**b**) CMF-HDTMS.

The FT-IR analysis was used to investigate the possible chemical interaction between the PLA and untreated or modified CMFs, and the results are given in Fig. 11. In the spectrum of neat PLA, absorption bands located at 2999, 2950, 1753, 1259, 1087, and 869/cm are corresponded to the vibrations of ν_asC–H_, ν_sC–H_, ν_C=O_, ν_COC_, ν_sCOC_, and ν_C–COO_, respectively^20^. According to the spectra of PLA composites incorporated with 5–30 wt% untreated CMF (Fig. 11a), no major differences can be seen between the chemical structure of neat PLA and its composites. The similarity between the FT-IR spectra of neat PLA and PLA-based composites incorporated with the untreated CMF suggests a lack of significant variations in their chemical structures. This observation could be attributed to the limited interfacial adhesion and chemical interaction between the polymer matrix and untreated CMF^36^. The FT-IR results are in line with the SEM morphological images of PLA/untreated CMF composites, which illustrate poor compatibility of PLA and CMF by the presence of voids around the fibers.

Figure 11b displays the FT-IR spectra of PLA-based composites reinforced with 5–30 wt% of CMF-HDTMS. As can be seen, a new peak appeared at 450/cm, which is assigned to the stretching vibrational of the Si–O–Si group of modified fibers. The intensity of typical silica bands (Si–O–Si stretching) at 1084/cm enhanced as the content of CMF-HDTMS was increased in composites. It should be noted that there is an overlap between the C–O–C stretching bond of PLA (at 1087/cm) and the band related to the Si–O–Si stretching (at 1084/cm) present in the modified fibers. Additionally, by the incorporation of CMF-HDTMS, the intensity of stretching vibration of the C–COO band (ν_C-COO_) at 869/cm decreased and a new band appeared at 1380/cm that is attributed to the deformational vibrations of C–H in the CH_3_ group of PLA. As mentioned in SEM results, it was found that the modification of CMF with HDTMS improved the dispersibility of fibers in PLA. It is assumed that the deposition of hydrophobic long-chain alkylsilanes onto the SiO_2_-coated CMF can improve their compatibility with the non-polar polymer matrix, thereby reducing the formation of voids and deterioration of the barrier performance of composites.

#### TGA test

**Figure 12:**
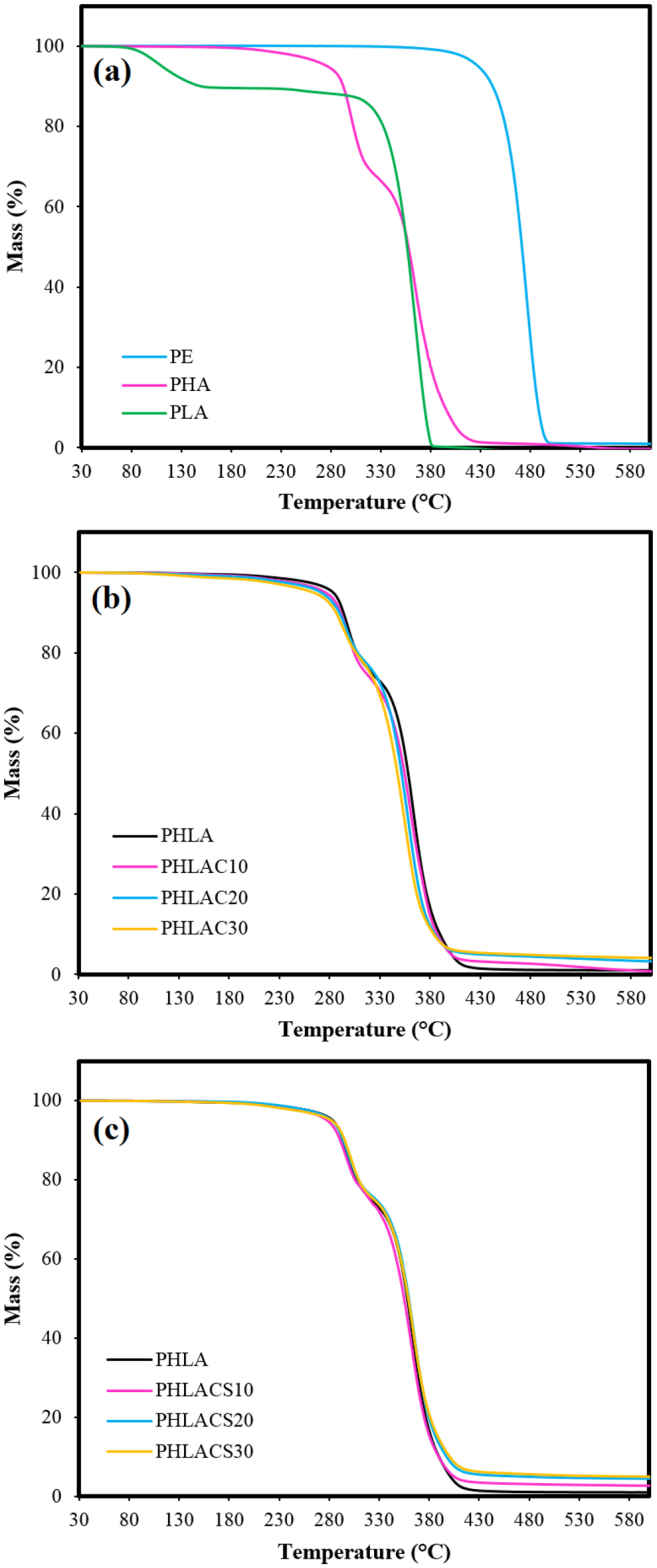
TGA curves of (**a**) neat PE, PHA, and PLA, (**b**) PHLACx, and (**c**) PHLACSx sandwich-structured composites.

In practice, thermal decomposition occurs as the properties of a polymeric material undergo changes under the influence of elevated temperatures. Indeed, thermal energy causes vibration in the chemical bond of polymers, and whenever this vibration exceeds the chemical bond strength, it leads to bond ruptures and thermal degradation of materials^37^. The thermal stability of prepared composites was determined using the TGA analysis and the results are presented in Fig. 12 and Table 3. Figure 12a displays the TGA curves of neat polymers including the commercial PE vapor barrier, PLA, and PHA. As shown, both PE and PLA were thermally degraded in a single step with maximum degradation temperatures (T_max_) of 477.2 and 365.9 °C, respectively. The thermal decomposition of PLA is due to the hydroxyl end-initiated ester interchange process and polymer chain hydrolysis^38^. The neat PHA exhibits a two-step decomposition process with two T_max_ located at 301.4 and 368.2 °C. The degradation reaction of PHA is attributed to the cleavage of ester groups by the elimination reaction^39^. The results illustrate that PE had a higher level of thermal stability in comparison with the neat PHA and PLA. This can be explained by the fact that the C–C bonds in PE are stronger than the ester groups in the biopolymers, thereby higher level of energy is required to break the C–C bonds of PE^40^.

**Table 3:** Thermal parameters of samples from the TGA analysis.

Sample	T_10_ (°C)	T_max1_ (°C)	T_max2_ (°C)	T_90_ (°C)
Untreated CMF	295.4	349.8	–	–
Modified CMF (CMF-HDTMS)	295.4	353.9	–	–
PE	442.2	477.2	–	488.2
PLA	149.4	365.9	–	374.1
PHA	293.8	301.4	368.2	394.7
PHLA	292.3	298.1	366.4	389.9
PHLAC10	291.1	298.2	364.4	387.2
PHLAC20	289.7	296.3	359.1	384.9
PHLAC30	286.3	292.7	355.7	383.7
PHLACS10	291.1	296.6	364.8	389.6
PHLACS20	294.1	299.2	367.3	397.5
PHLACS30	295.6	301.5	367.8	400.6

The TGA results of sandwich composites (Fig. 12b and c) indicate a thermal degradation pattern similar to that of neat PHA, with the absence of the initial decomposition temperature (T_10_) of PLA in the composites. This might be attributed to their composition, consisting of approximately 66% PHA in the surface layers and 33% PLA in the interlayer, resulting in a thermal degradation pattern of sandwich composites closely mirroring that of neat PHA. Nevertheless, for the PHLA composite, a closer look at Table 3 reveals that a slight reduction in thermal decomposition temperatures at T_10_, T_max1_, T_max2_, and T_90_ is evident, highlighting the impact of the PLA interlayer (having a lower thermal stability than PHA) on the thermal behavior of sandwich composite. In the case of PHLACx sandwich composites, it can be observed that the addition of untreated CMF decreased the thermal stability of composites. For instance, by the incorporation of 30% untreated fibers into the sandwich composite (PHLAC30), the values of T_10_, T_max2_, and T_90_ were reduced by 2, 3, and 1.6%, respectively. On the contrary, the PHLACSx sandwich composites showed improvement in thermal stability, indicating the reinforcement effect of the CMF-HDTMS. Interestingly, the PHLACS30 showed the highest values of T_10_ (295.6 °C), T_max2_ (367.8 °C), and T_90_ (400.6 °C) among all composites. It can be expected that the addition of highly thermal stable CMF-HDTMS acted as an efficient heat sink in the structure that could extract more heat than the polymer matrix and protect the materials from thermal degradation^35^. Similarly, Botta et al.^41^ demonstrated that the addition of highly thermal stable graphene particles into PLA improved the thermal stability of composites. Therefore, it could be concluded that the incorporation of sol–gel modified fibers into sandwich composites had a significant impact on improving the thermal stability of materials since the higher level of activation energy required for the thermal decomposition of the composites^42^.

#### Tensile test

##### Mechanical properties before the accelerated aging

The mechanical property is a crucial aspect of vapor barrier membranes, directly influencing their functionality and long-term performance within building envelope applications. Therefore, when specifying materials for this application, careful consideration of the mechanical characteristics of barrier membranes is essential. The barrier membrane should possess sufficient flexibility to be foldable, allowing it to move with the building and effectively cover the walls^43^. In this regard, the tensile test (according to the ASTM D882 standard) was carried out to analyze the mechanical properties of developed membranes. The mechanical characteristics of the commercial PE barrier membrane, PHA, PLA, and sandwich-structured composites are provided in Table 4. The PE membrane had tensile strength and elongation at break values of 19.1 ± 1.4 MPa and 337.2 ± 12.8%, respectively. The tensile strength value of both neat biopolymers (PHA and PLA) was around 23 MPa, while they showed lower values of elongation at break compared to the PE membrane. These values are in line with the previous research studies^44^. Generally, it is known that the poor mechanical properties of PLA and PHA are due to their low crystallinity and slow crystallization behavior^45^.

**Table 4:** The results of tensile tests of samples.

Sample	Young’s modulus (MPa)	Tensile strength (MPa)	Elongation at break (%)
PE	113.1 ± 13.7	19.1 ± 1.4	337.2 ± 12.8
PHA	1557.9 ± 85.2	24.3 ± 0.1	2.3 ± 0.3
PLA	2511.2 ± 78.8	23.4 ± 0.8	37.5 ± 1.3
PHLA	2221.3 ± 348.6	21.2 ± 1.2	1.3 ± 0.1
PHLAC5	2334.8 ± 138.1	22.6 ± 1.9	1.3 ± 0.2
PHLAC10	2072.6 ± 158.2	21.5 ± 1.8	1.3 ± 0.1
PHLAC20	2273.8 ± 90.9	22.9 ± 3.7	1.2 ± 0.3
PHLAC30	2588.6 ± 434.3	25.9 ± 5.2	1.3 ± 0.4
PHLACS5	2767.2 ± 21.6	29.1 ± 1.3	1.3 ± 0.1
PHLACS10	2359.2 ± 71.8	22.2 ± 1.4	1.1 ± 0.1
PHLACS20	2663.9 ± 272.6	22.45 ± 2.3	1.2 ± 0.2
PHLACS30	2706.8 ± 298.4	25.6 ± 2.6	1.3 ± 0.1

The PHLA sandwich composite with the interlayer of PLA showed slightly lower values of tensile strength (21.2 ± 1.2 MPa) and elongation at break (1.3 ± 0.1%) in comparison with the neat PHA. This result might be due to the relatively weak attachment between the layers of composites. However, the PHLA composite had a higher value of Young’s modulus (2221.3 ± 348.6 MPa) than the neat PHA. It is worthy to note that the mechanical properties of sandwich-structured composites depend on material properties of each layer, and the strength of interface between the layers of composites^46^. Samples with the interlayer of PLA-based composites showed elongation at break values of ~ 1.3%, which demonstrates that the incorporation of either untreated CMF or CMF-HDTMS into the PLA interlayer had no evident effect on the flexibility of sandwich composites. Interestingly, the PHLACx and PHLACSx sandwich composites exhibited higher values of Young’s modulus and tensile strength compared to the PHLA composite, suggesting a reinforcing effect imparted by cellulose fibers. This observation aligns with a previous research study, which reported that PLA-based composites reinforced with regenerated cellulose demonstrated an enhancement in Young’s modulus and tensile strength values^47^. As can be observed in Table 4, a higher improvement of mechanical properties was observed in the case of PHLACSx sandwich composites compared to PHLA and PHLACx composites. For instance, the PHLACS5 composite, in which the PLA-based interlayer was incorporated with 5 wt% of CMF-HDTMS, exhibited the highest values of Young’s modulus (2767.2 ± 21.6 MPa) and tensile strength (29.1 ± 1.3 MPa) among all samples. These values were respectively 24.5% and 37.2% higher that of the PHLA composite. The results demonstrate a significant impact of sol–gel modification of CMF on the mechanical properties of the composites. This is attributed to the improved dispersibility of CMF-HDTMS within the polymer matrix, leading to enhanced adhesion between fibers and the polymer matrix at the interface. Additionally, according to the obtained results, the PHLACSx sandwich composites displayed superior values of Young’s Modulus and tensile strength compared to the commercial PE barrier membrane. Furthermore, the flexibility and foldability of PHLACSx sandwich composites make them suitable for effectively covering building walls. This suggests their potential candidacy for the development of barrier membranes in building envelope applications.

**Figure 13:**
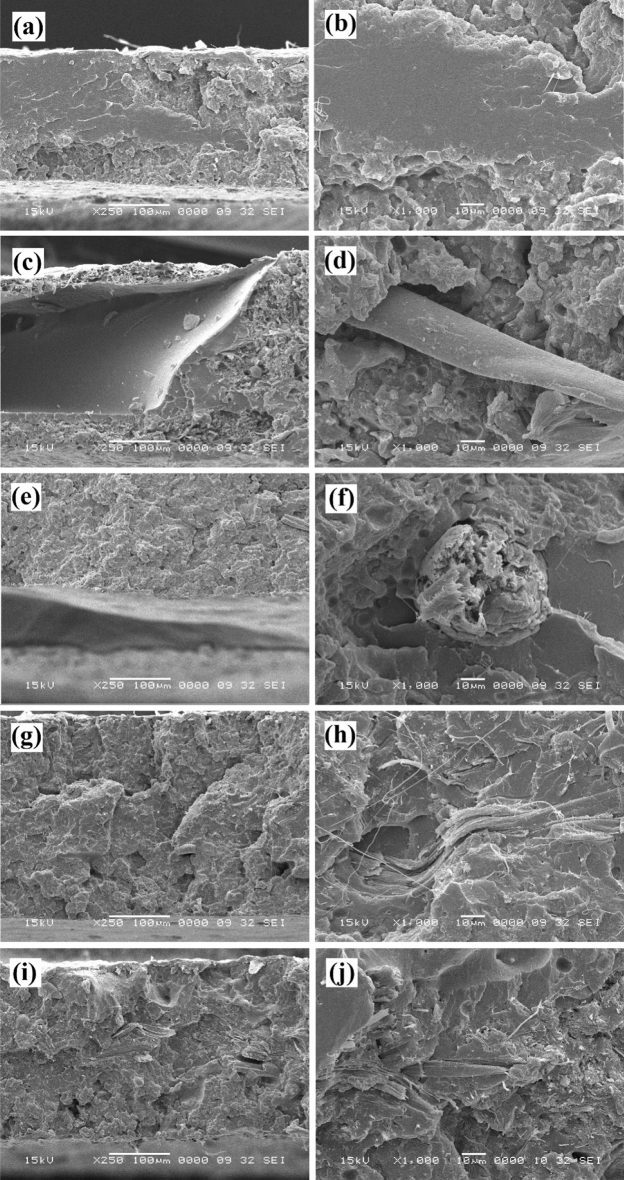
SEM images of the tensile fractured surface of: (**a** and **b**) PHLA, (**c** and **d**) PHLAC5, (**e** and **f**) PHLAC10, (**g** and **h**) PHLACS5, and (**i** and **j**) PHLACS10.

The SEM images of the tensile fracture surface of sandwich composites are presented in Fig. 13. The fracture in polymers takes place by two different mechanisms depending on their brittle or ductile behavior^48^. Generally, the brittle fracture requires less energy than ductile fracture and involves fracture without any appreciable plastic deformation (elastic behavior). The topography of brittle fracture appears smooth under the SEM analysis. The ductile fracture usually requires a higher level of energy and involves large plastic deformation before the rupture^49^. Figures 13a and b exhibit the tensile fracture surface of the PHLA composite. As can be observed in these figures, the PLA interlayer has a smooth morphology, whereas the PHA surface layers showed a plastic deformation and irregular topography. In addition, there is a proper attachment between the PHA and PLA layers at the fracture surface of the PHLA composite. Indeed, the attachment between layers in a multi-layer composite is very essential since it plays an important role in transferring the applied stress between the layers of composites^38^.

The tensile fracture surface of PHLAC5 and PHLAC10 sandwich composites are given in Fig. 13c–f. A closer look at these SEM images reveals that the interlayers, which are the PLA incorporated with the untreated CMF, were separated, and pulled out from the structure after the tensile failure. This might be due to the difference in polarity between the layers of sandwich composites. PHA possesses a non-polar structure, whereas the presence of hydrophilic CMF in the PLA matrix could increase the polarity of the interlayer. Consequently, defects and discontinuity are established between the incompatible layers of sandwich composites that could negatively affect the mechanical properties of composites^50,51^. Moreover, the presence of fiber aggregates and voids around fibers is evident in the fracture surface of PHLAC5 and PHLAC10 composites (Fig. 13d and f), indicating low compatibility between the untreated CMF and the polymer matrix. The poor dispersibility of fibers and their agglomeration in the polymer matrix led to a non-uniform load distribution on the materials, thereby resulting in deformation at an early stage of tensile testing^52^. Figure 13g–j shows that the CMF-HDTMS was well dispersed in the interlayers of PHLACS5 and PHLACS10 sandwich composites, without the presence of any voids or fiber aggregates at their fracture surfaces. Additionally, no separation can be observed between the layers of sandwich composites after the rupture which implies a good attachment between the PHA surface layers and PLA/CMF-HDTMS interlayer. Consequently, it can be inferred that the hydrophobic modification of CMF with HDTMS (via the sol–gel process) effectively enhances fiber dispersibility in the polymer matrix and plays a significant role in heightening the interfacial compatibility between the layers of PHLA sandwich composites.

##### Mechanical properties after the accelerated aging

Material durability has recently gained attention in the national building code of Canada (NBCC) to determine the appropriate quality level for the building envelope materials. It has been reported that the assumed lifespan of PE sheeting is between 2 and 15 years in the vapor barrier membrane application^53^. These membranes are in contact with several internal sources of moisture (e.g., showers, taps, and cooking) during their service lifetimes, leading to degradation and deterioration of their properties. The degradation of polymeric materials by moisture can be explained by the hydrolysis and reaction of water molecules that leads to the cleavage of functional groups of polymers. In semi-crystalline polyesters (e.g., PHA and PLA), the hydrolysis occurs in two stages; (1) diffusion of water molecules into the amorphous regions of polymer and (2) the moisture penetration and degradation causing cleavage of polymer chains^54^.

**Figure 14:**
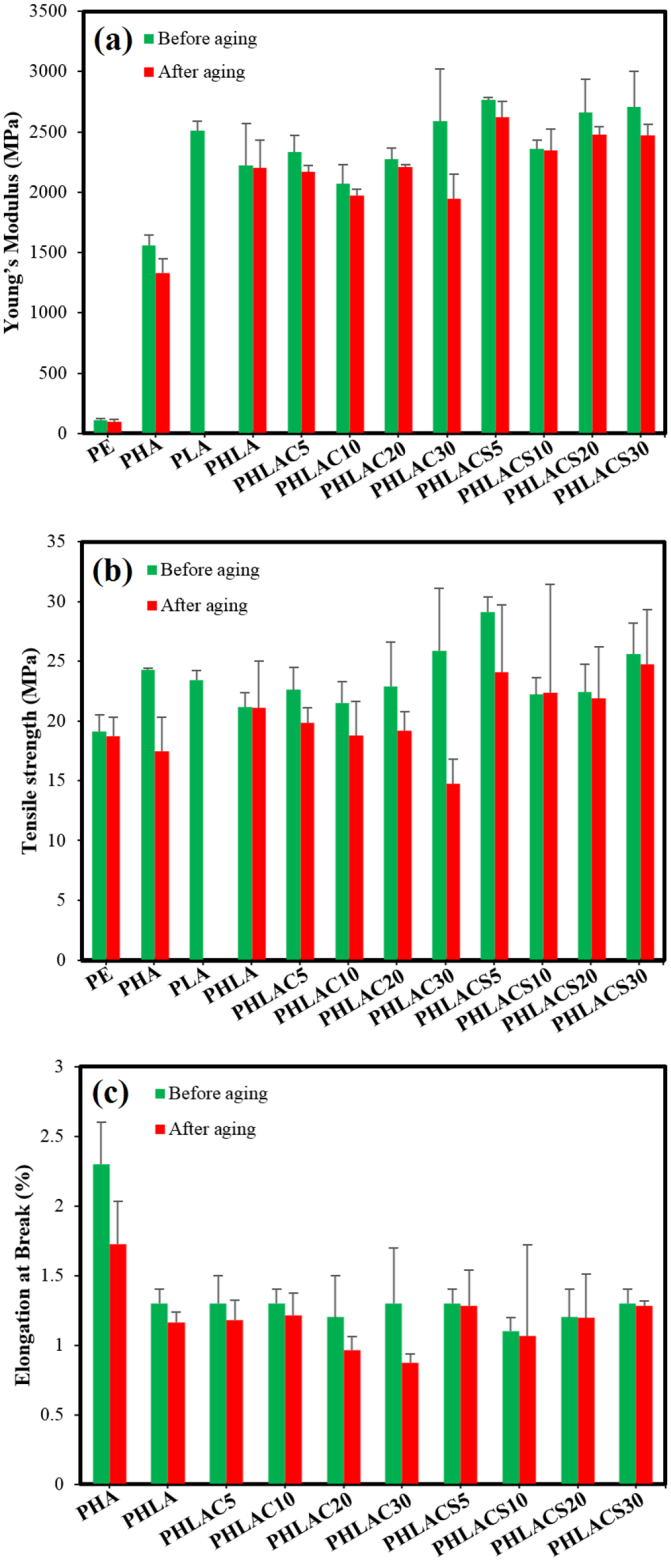
The mechanical properties of sandwich-structured composites before and after the accelerated aging process: (**a**) Young’s modulus, (**b**) tensile strength, and (**c**) elongation at break.

The durability test was performed according to the ASTM D1183 standard to determine the effect of accelerated aging on the mechanical properties of sandwich-structured composites. In this regard, the samples were exposed to 10 cycles of high (90% RH) and low (25% RH) humidity over 40 days. Subsequently, the investigation of mechanical properties was conducted to evaluate the effects of the accelerated aging process. Figure 14 illustrates the results of the tensile test before and after the accelerated aging. Overall, it is evident that the mechanical characteristics of materials deteriorated after the accelerated aging process. For instance, the tensile strength values for the PE membrane and neat PHA were reduced by 2% and 28.1%, respectively. Moreover, the values of elongation at break of aged PE and PHA were 311.6 ± 13.4% and 1.7 ± 0.3%, representing a 7% and 25% reduction compared to their respective values before the accelerated aging process. The observed results are due to the absorption of moisture by the materials during the durability test, leading to polymer hydrolysis. This process causes a reduction in molecular weight due to random chain scission of polymer backbones, ultimately resulting in polymer degradation^55^. It is crucial to know that the mechanical properties of polymers are extremely sensitive to molecular weight, and therefore chain scission has a significant impact on the mechanical performance of polymers^56^. Similarly, Philip et al.^57^ investigated the effect of weathering in polymer products and demonstrated that the reduction of polymer molecular weight, attributed to chain scission, resulted in shorter polymer chains, consequently leading to a decline in the mechanical properties of the materials. It should be noted that there is no available data for the neat PLA after the accelerated aging since the samples were folded and very brittle to be tested without problems. This might be due to the rearrangement of polymer chains by exposing them to moisture and cyclic humidity conditions^58^.

According to Fig. 14, accelerated aging had a more negative effect on the mechanical properties of PHLACx composites in comparison to the PHLA composite. This might be because of the presence of highly hydrophilic CMF in the composites that could absorb and hold moisture from the surrounding environment, consequently, facilitating the chain scission of PHA and PLA biopolymers. For instance, the lowest mechanical properties were observed in the case of PHLAC30, in which the Young’s modulus, tensile strength, and elongation at break values decreased by 24.9%, 43.1%, and 32.9%, respectively, after the accelerated aging process. Similar results were observed by Abdul Qadeer Dayo et al., who investigated the effect of accelerated weathering on the mechanical properties of polybenzoxazine composites reinforced with the hemp fibers. The results demonstrated that the mechanical characteristics of composites extremely declined after weathering test, which is mainly due to the absorbed moisture by the hemp fibers in the structure^59^. Interestingly, a closer look at Fig. 14 illustrates that the PHLACSx sandwich composites showed the minimum changes in the mechanical properties after the accelerated aging process, and the mechanical characteristics of aged PHLACSx composites were higher than that of aged PHLACx composites. For example, the values of Young’s modulus, tensile strength, and elongation at break of aged PHLACS30 composites were 2473.5 MPa, 24.7 MPa, and 1.2%, respectively, which were slightly reduced by 8.6%, 3.2%, and 1.6% after the accelerated aging. The observed results can be attributed to the hydrophobic modification of CMF via the sol–gel process, which reduced the absorbed moisture by fibers during the durability test. Consequently, sandwich composites reinforced with CMF-HDTMS experienced the least deterioration in their mechanical properties.

#### WVTR test

##### Barrier performance before the accelerated aging

The barrier performance against water vapor and moisture is the most important characteristic of plastic membranes in the building envelope application. In cold climate countries, assemblies require protection from the interior moisture. Therefore, a vapor barrier membrane should be installed towards the interior side (warm side) of building envelopes to control the migration of moisture that otherwise leads to mold growth, moisture accumulation, and deterioration of materials. The diffusion rate of water vapor is a function of the vapor permeance of the building envelope material and the magnitude of water vapor pressure difference across the barrier membrane^1^. Generally, the migration of moisture through the building envelope arises from two mechanisms; (1) convective moisture transfer, which includes the process of diffusion of water vapor and moisture through the building wall components, and (2) advective transfer of moisture, which is because of the air movement through the openings in the building wall assembly. It is worth noting that several parameters affect the barrier performance of plastic membranes such as the crystallinity of polymers, the hydrophobic/hydrophilic nature of materials, the presence of additives in the polymer matrix, and porosity^60^.

**Figure 15:**
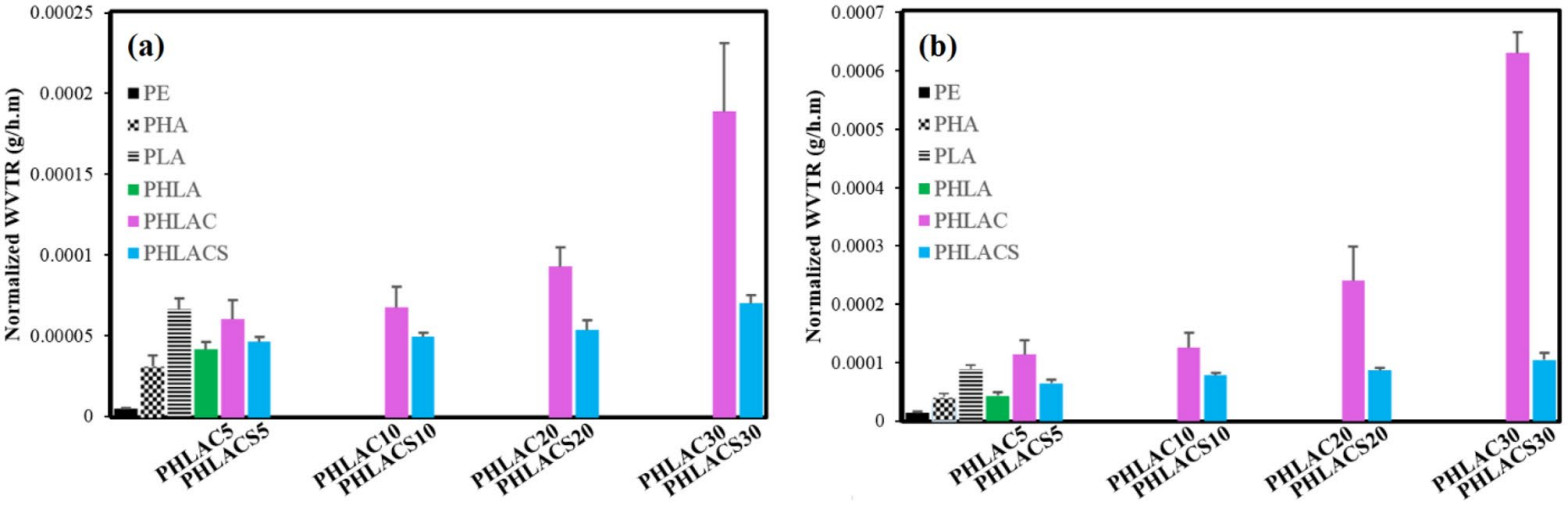
The normalized WVTR values (**a**) desiccant method and (**b**) water method of sandwich-structured composites before the accelerated aging process.

The vapor barrier performance of materials, including the commercial PE membrane, neat biopolymers (PHA and PLA), and developed sandwich-structured composites was investigated by the water vapor transmission rate test (WVTR). In this regard, the WVTR test was performed based on the ASTM E96 standard for both desiccant (method A) and water (method B) methods, and the obtained results are presented in Fig. 15 and Table 5. The values of normalized WVTR (water method) for the commercial PE membrane, neat PHA, and PLA are about 1.37 × 10^–5^, 4.1 × 10^–5^, and 8.9 × 10^–5^ g/h m, respectively. The PE membrane exhibited superior barrier properties compared to the neat biopolymers. This superiority is due to the PE's inherently non-polar character and high density, contributing to its exceptional moisture barrier performance over PHA and PLA^54^. In addition, the neat PHA showed a higher vapor barrier performance in comparison with the neat PLA, which considered the selection of PHA for the surface layers of sandwich composites in this study. Previous studies demonstrated that PHA has a similar barrier performance to that of fossil-based thermoplastics such as polyvinyl chloride or polyethylene terephthalate, making it an attractive candidate in several applications such as food packaging and vapor barrier membrane applications^61^.

**Table 5:** The normalized WVTR values of sandwich-structured composites before and after the accelerated aging process.

Sample	Normalized WVTR (g/h m) × 10^5^			
	Desiccant method (method A)		Water method (method B)	
	Before aging	After aging	Before aging	After aging
PE	0.5 ± 0.1	0.54 ± 0.1 (↑8%)	1.4 ± 0.3	1.56 ± 0.2 (↑ 11%)
PHA	3.1 ± 0.7	3.8 ± 0.4 (↑ 22%)	4.1 ± 0.6	5.2 ± 0.5 (↑ 26%)
PLA	6.7 ± 0.7	9.1 ± 1.8 (↑ 35%)	8.9 ± 0.6	12.5 ± 2.4 (↑ 40%)
PHLA	4.2 ± 0.5	5.3 ± 0.6 (↑ 26%)	4.3 ± 0.7	5.7 ± 2.4 (↑ 32%)
PHLAC5	6.1 ± 1.2	10.6 ± 2.3 (↑ 73%)	11.4 ± 2.4	21.6 ± 7.1 (↑ 89%)
PHLAC10	6.8 ± 1.3	14.1 ± 3.1 (↑ 107%)	12.5 ± 2.4	27.4 ± 3.4 (↑ 119%)
PHLAC20	9.3 ± 1.2	21.6 ± 1.8 (↑ 132%)	23.9 ± 5.8	60.7 ± 3.3 (↑153%)
PHLAC30	18.8 ± 4.2	45.1 ± 3.4 (↑ 139%)	62.7 ± 3.6	168.6 ± 13.6 (↑ 168%)
PHLACS5	4.7 ± 0.3	6.1 ± 0.3 (↑ 29%)	6.5 ± 0.6	8.6 ± 0.7 (↑ 32%)
PHLACS10	4.9 ± 0.2	6.7 ± 0.3 (↑ 36%)	7.8 ± 0.3	10.4 ± 2.6 (↑ 33%)
PHLACS20	5.4 ± 0.6	6.9 ± 0.9 (↑ 40%)	8.7 ± 0.3	12.1 ± 5.4 (↑ 39%)
PHLACS30	7.1 ± 0.5	9.7 ± 1.3 (↑ 36%)	10.4 ± 1.2	15.3 ± 2.1 (↑ 47%)

The normalized WVTR value (water method) of the PHLA composite was 4.3 × 10^–5^ g/h m, which is somewhat higher than the neat PHA. This could be attributed to the presence of the PLA interlayer having a lower barrier performance than the neat PHA. As shown in Fig. 15a and b, the barrier performance of PHLACx sandwich composites was remarkably reduced by the addition of untreated CMF into the PLA interlayer. The PHLAC30 exhibited the lowest barrier performance among all samples, which has the normalized WVTR (method B) value of ~ 62.7 × 10^–5^. The decrement in barrier performance is due to the hydrophilic nature of untreated CMF that could absorb moisture from the surrounding environment and facilitate the migration of water vapor through the membrane. Furthermore, another contributing factor could be the poor compatibility between the untreated fibers and polymer matric, resulting the formation of voids and fiber aggregates within the structure. These defects create a preferential pathway for the migration of water vapor molecules, thereby negatively affecting the barrier performance of the materials^60^.

Interestingly, the PHLACSx composites showed superior barrier performance and had significantly lower values of normalized WVTR (for both methods A and B) than the PHLACx composites. The values of normalized WVTR (water method) for PHLACSx, where the PLA interlayer was incorporated with 5–30 wt% of CMF-HDTMS, ranged from ~ 6.5 × 10^–5^ to 10.4 × 10^–5^ g/h m, which are notably comparable to that of the PHLA composite (4.3 × 10^–5^ g/h m). These results could be explained mainly by two reasons: (1) the highly hydrophobic characteristic of CMF after the sol–gel modification (as shown in the contact angle test results), and (2) the enhanced dispersibility of modified fibers in the polymer matrix that reduced the formation of fiber aggregates, voids, and structural defects. These obtained results emphasize the significant effect of sol–gel modification of CMF in improving the barrier performance of developed sandwich composites against water vapor.

##### Barrier performance after the accelerated aging

The normalized WVTR values of developed membranes after the accelerated aging process are presented in Table 5. It is evident that the accelerated aging resulted in a reduction in the barrier performance of the materials, attributed to the hydrolysis of polymer chains occurring during the durability test. The increase percentages of the normalized WVTR values of all samples are indicated in Table 5. It is worth indicating that the PE membrane showed a lower reduction of barrier performance than the neat biopolymer (PHA and PLA). This might be attributed to the difference in the chemical functional groups of polymers. The carbonyl and carboxyl -COOH groups of PHA and PLA can interact with the absorbed water molecules, thereby leading to being affected more by the hydrolysis of polymer chains^61^.

According to Table 5, the PHLACx sandwich composites, where the interlayer was incorporated with the untreated CMF, exhibited lower values of vapor barrier properties in comparison with the PHLA composite. As mentioned earlier, cellulose fibers have hygroscopic characteristics that can attract and hold moisture from the surrounding environment, consequently, facilitating the hydrolysis of polymer chains during the accelerated aging process. Hydrolysis of the ester groups of PHA and PLA biopolymers changes high-molecular-weight to low-molecular-weight polymers. As a result, a large number of carboxyl and hydroxyl end groups are formed in aged polymers. These functional groups can interact with the water vapor molecules and ease the diffusion of water vapor into the materials^62^. Furthermore, it should be noted that each polymer chain end group contributes to some free volume, and an aged polymer, after the durability test with shorter chains (lower molecular weight), has more chain ends per unit volume. Consequently, the hydrolysis of polymers is expected to increase the free volume in materials, which is favorable for the migration of moisture through the membranes^63^. Interestingly, the PHLACSx sandwich composites showed superior barrier performance than that of PHLACx composites after the durability test. This observation confirms the hydrophobic modification effect of CMF that has been previously explained in the tensile test results. Indeed, the presence of hydrophobic long-chain alkylsilanes onto the CMF’s surface could effectively reduce the absorbed moisture by fibers, thus restricting the dramatic hydrolysis of polymer chains during the accelerated aging process.

According to the NBCC standard, a barrier membrane installed on the interior side of the building wall must have a water vapor permeance value of less than 60 ng/Pa s m^2^ for indoor spaces maintained below 35% relative humidity, which is equal to the normalized WVTR value of ~ 9.1 × 10^–5^ g/h m (for a membrane with a thickness of 300 µm)^64^. Table 5 demonstrates that the PHLACSx sandwich composites had the normalized WVTR values (desiccant method) of ~ 6 × 10^–5^ to 9 × 10^–5^ g/h m after the accelerated aging process, indicating that PHLACSx could be considered appropriate candidates for the development of vapor barrier membranes in building envelope applications.

## Conclusion

This research study aims to develop a sandwich-structured barrier membrane from bio-based materials, including polyhydroxyalkanoate (PHA), polylactic acid (PLA), and cellulose microfibers (CMF). The barrier membranes used in the building envelope can control the migration of water vapor through the building walls that otherwise lead to moisture accumulation and deterioration of building materials. In this regard, multilayer composites were fabricated from the PHA sheets (surface layers) and fibers reinforced PLA composites (interlayers) by the compression molding technique. In order to improve the dispersibility of CMF in PLA, the surface of fibers was chemically modified via a sol–gel process. Through this modification method, the spherical silica nanoparticles (SiO_2_) were formed on the surface of CMF, thereafter the hydrophobic long-chain alkylsilane was chemically bonded onto the SiO_2_ nanoparticles. The results demonstrated that the hydrophobicity and thermal stability of CMF remarkably enhanced after the sol–gel modification. The morphological investigations showed that the modified fibers (CMF-HDTMS) uniformly dispersed in the PLA matrix without significant agglomeration. Moreover, the prepared sandwich composites were characterized for their thermomechanical and vapor barrier properties through the thermogravimetric analysis (TGA), tensile test, and water vapor transmission rate (WVTR) analysis. The TGA test results indicated that the thermal stability of materials improved thanks to the presence of CMF-HDTMS in the composites. The tensile test results showed that the tensile strength and Young’s modulus of materials increased by the incorporation of CMF-HDTMS into the composites. Regarding the WVTR test, the barrier performance of composites reduced as the untreated CMF content increased in materials; however, the composites reinforced with CMF-HDTMS showed superior barrier performance. Additionally, the decrement of mechanical and barrier properties was observed when the materials were exposed to the accelerated aging process, while the sandwich composites reinforced with CMF-HDTMS experienced the least deterioration in their properties. In conclusion, integrating biopolymers (PHA and PLA) into a sandwich-structured membrane, along with sol–gel modified fibers, offers a promising solution for the fabrication barrier membranes in building envelopes. This success signifies the potential of biopolymers as alternatives to conventional petroleum-based materials in construction applications. Moreover, further research could be conducted to explore both the biodegradability of prepared bio-based membranes after their service life and the development of effective waste management strategies. Simultaneously, additional studies are warranted to enhance the flexibility of these composites for broader applications.

## Data Availability

All data in this study will be available from the corresponding author on reasonable request.
